# Turner Syndrome With 45,X/46,XX Mosaicism and a Derivative X Chromosome (Xqter → Xq13?::Xp11.4 → Xqter): A Case Report

**DOI:** 10.1002/ccr3.70981

**Published:** 2025-09-28

**Authors:** Weijun Jiang, Tingting Ji, Qiuyue Wu, Zhipeng Xu, Xinyi Xia

**Affiliations:** ^1^ Institute of Laboratory Medicine Jinling Hospital, Affiliated Hospital of Medical School, Nanjing University Nanjing Jiangsu China; ^2^ State Key Laboratory of Analytical Chemistry for Life Science Nanjing University Nanjing Jiangsu China; ^3^ Jinling Hospital, The First School of Clinical Medicine, Southern Medical University Nanjing Jiangsu China; ^4^ Department of Reproductive Medicine Suzhou Dushu Lake Hospital, The Fourth Affiliated Hospital of Soochow University Suzhou Jiangsu China

**Keywords:** chromosomal microarray, derivative X chromosome, MLPA, mosaicism, turner syndrome

## Abstract

Chinese female with 45,X/46,XX mosaicism and der(X) (Xqter → Xq13?::Xp11.4 → Xqter). *SHOX* deletion (41.25 Mb) and *PLP1* duplication (111.4 Mb) linked to growth/neuro risks. Integrated karyotyping, MLPA, microarray, and STR analysis revealed cryptic X‐chromosome rearrangements, guiding precise breakpoint mapping. Multidisciplinary management (hormone therapy, cardiac monitoring, fertility counseling) is essential for optimizing outcomes in complex TS cases.

**Summary**

- Turner syndrome with rare X‐chromosome mosaicism (e.g., 45,X/46,XX,der(X)) necessitates integrative genetic testing (karyotyping, MLPA, microarray) to resolve diagnostic discrepancies.
- Clinicians must prioritize multidisciplinary care addressing gonadal failure, cardiovascular risks, and neurodevelopmental monitoring.
- Early hormone replacement and lifelong surveillance are critical for optimizing outcomes.

## 1 Introduction

Turner syndrome (TS) is well known as one of the most common chromosomal aberration disorders [1, 2]. It is a congenital disease caused by numerical (e.g., monosomy X) or structural aberrations (e.g., isochromosomes, deletions) of sex chromosomes, occurring in approximately 1:2000–1:2500 live female births [2, 3, 4, 5]. Mosaic forms, such as 45,X/46,XX, may present with attenuated phenotypes, including partial ovarian function or milder somatic features, depending on the proportion and distribution of abnormal cell lines. It is usually characterized by short stature, dysmorphic features, endocrine disturbances, and gonadal dysgenesis, resulting in delayed puberty, primary amenorrhea, and infertility [4]. Spontaneous puberty is observed in 15%–30% of girls with TS, and 2%–20% of patients experience menarche without hormonal substitution [6]. Spontaneous conceptions occur in 2%–8% [7]. Pregnancies in TS women are associated with an increased risk of poor outcomes both for mother and fetus [8].

Jacobs et al. first described a rare case with the 47/XXX karyotype in 1959 [9]. While the phenotypic characteristics of the 45X have been widely described, reports on the specific mosaicism are less frequent. Molecular studies comparing proband and parental X polymorphisms have shown that the maternal X chromosome is retained in about two‐thirds of subjects with Turner's syndrome and the paternal X in the remaining one‐third [8, 10]. The more frequent is the presence of an isochromosome of the long arm of the X (i(Xq)) and ring X and mosaicism for two or more normal or abnormal cell lines (e.g., 45,X/46,XX; 45,X/46,XY) [11]. A small proportion of 3%–4% of subjects with Turner syndrome is mosaic for a triple X (47/XXX) cell line [11, 12].

In the present report, we reviewed the published literature describing TS. Here, we first report a female chromosome karyotype: 45,X/46,XX,der(X)(Xqter → Xq13?::Xp11.4 → Xqter), mosaic ratio of 34/166, diagnosed as TS. All procedures adhered to the ethical standards of Jinling Hospital and the Helsinki Declaration. The patient provided written informed consent for anonymized data publication.

## 2 Case Report

### 2.1 Clinical Presentation

A 24‐year‐old Chinese female presented to the Department of Gynecology at Nanjing Jinling Hospital with primary amenorrhea and growth retardation. She was born at term to non‐consanguineous parents, with no family history of chromosomal disorders. Both parents and her younger brother exhibited normal development. Clinical examination revealed short stature (141 cm), a slightly webbed neck, elbow eversion, breast hypoplasia, and absence of axillary or pubic hair. Pelvic ultrasonography identified an immature left ovary, while the right ovary and uterus were not visualized.

### 2.2 Multidisciplinary Evaluation

Hormonal profiling showed elevated follicle‐stimulating hormone (FSH: 38.1 IU/L) and low estradiol (E_2_: 79.00 pmol/L), consistent with ovarian dysgenesis. Cardiac evaluation (echocardiography, blood pressure) revealed no structural anomalies. Renal function and bone metabolism markers (calcium, vitamin D) were within normal ranges. No evidence of autoimmune disorders or tumors was detected.

## 3 Genetic Analysis

### 3.1 Karyotyping and G‐Banding

**FIGURE 1:**
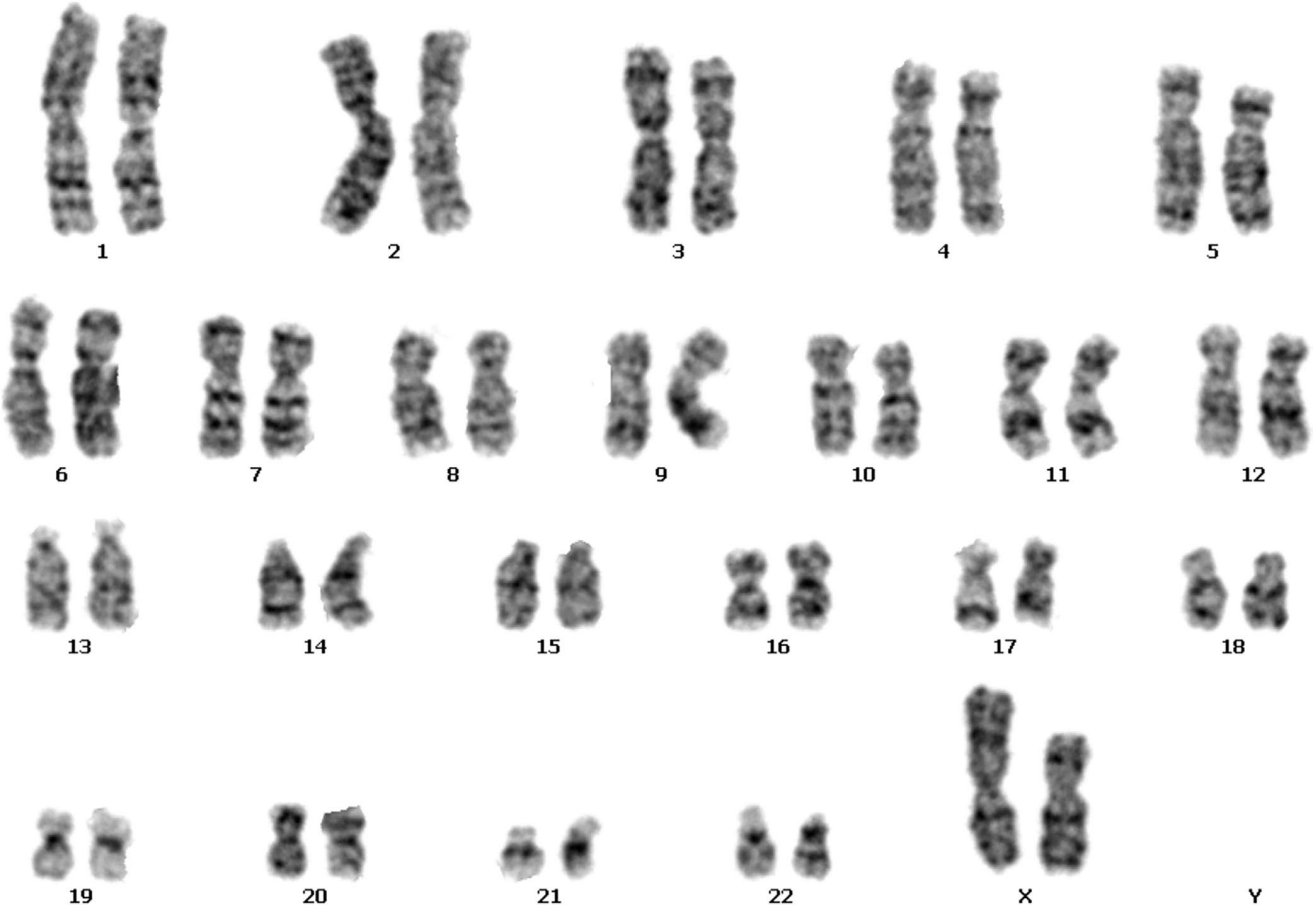
G‐banded karyotype of the derivative X chromosome (der(X)). Arrow indicates the der(X) with breakpoints at Xq13 and Xp11.4.

Conventional karyotyping of 200 metaphase cells (50 fully analyzed) revealed mosaicism: 45,X (34 cells) and 46,XX,der(X)(Xqter → Xq13?::Xp11.4 → Xqter) (166 cells) (Figure 1). The derivative X chromosome (der(X)) was characterized by breakpoints at Xq13 (long arm) and Xp11.4 (short arm), resulting in duplication of Xp11.4 → Xqter and deletion of Xq13 → Xp11.4.

### 3.2 Molecular Confirmation

**FIGURE 2:**
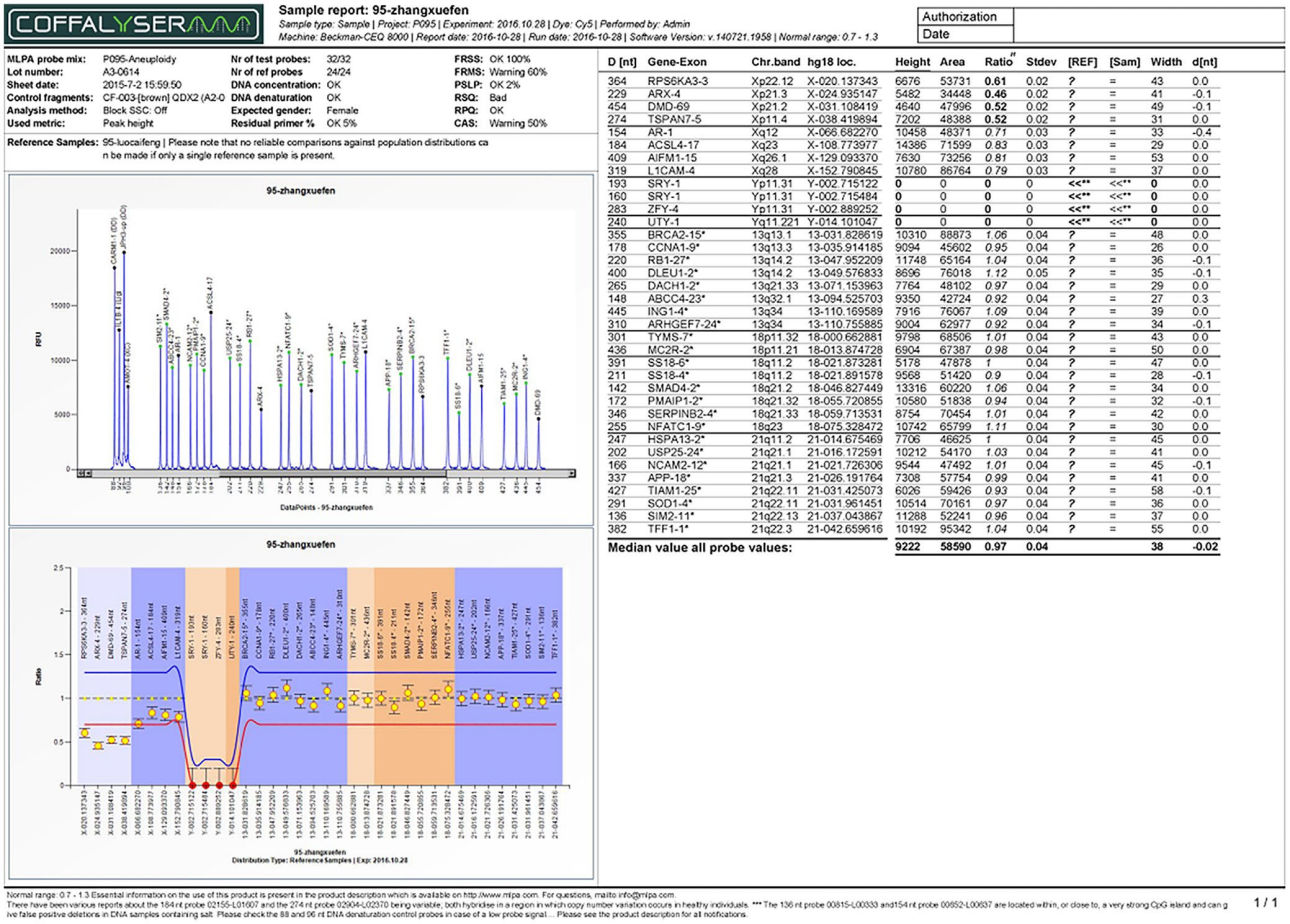
MLPA results confirming Xp deletion/Xq duplication. A female has one copy of all targets from Xp22.12 to Xp11.4, within the P095 kit [MLPA Xp22.12 to Xp11.4 (P095) × 1].

**FIGURE 3:**
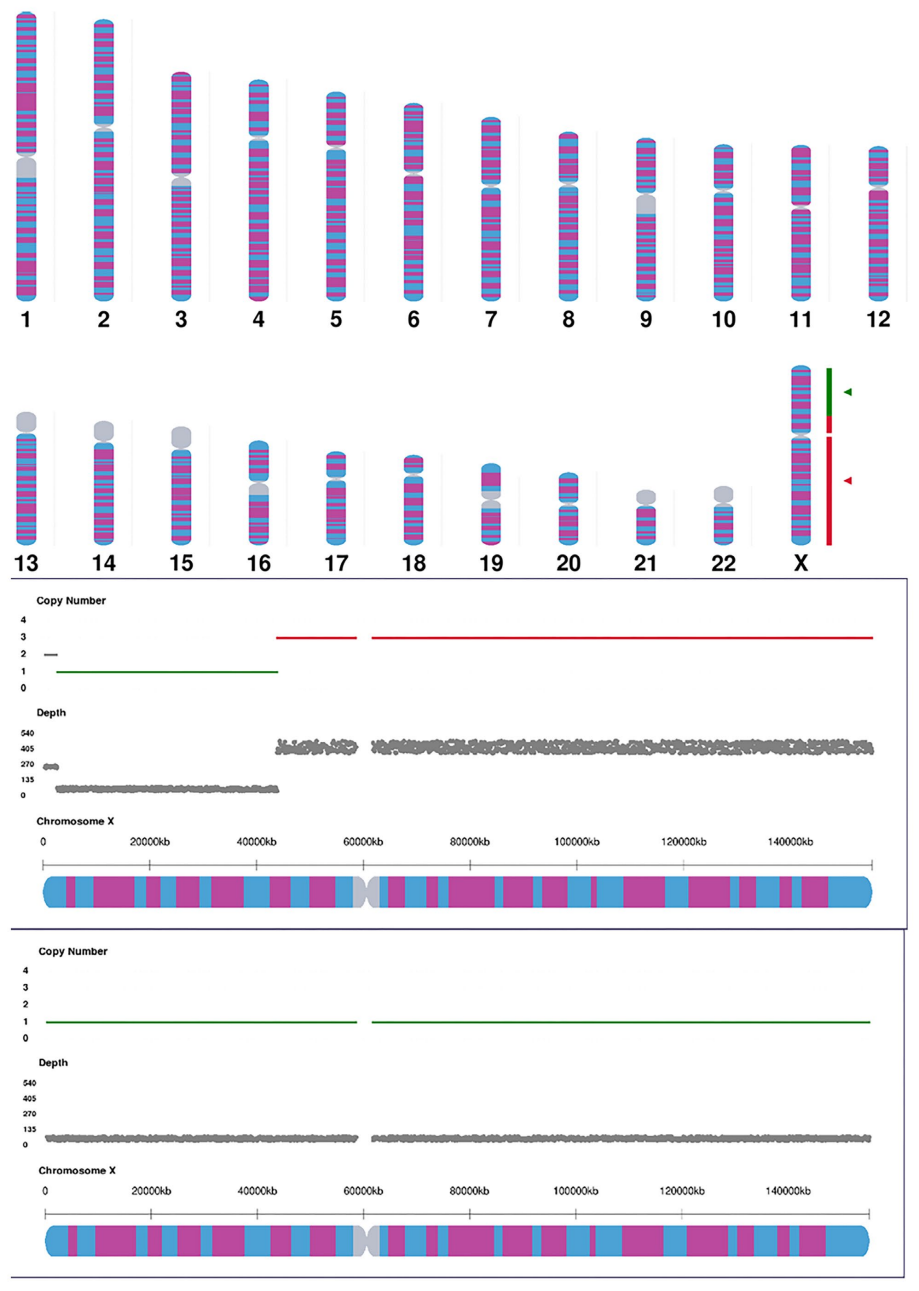
Chromosomal microarray results showing a 41.25 Mb deletion (Xp22.33‐p11.3, red) and 111.4 Mb duplication (Xp11.3‐q28, green). The red fragment represents that there are duplicates in this region. The green fragment represents the absence of the region. The gray fragment represents an area that cannot be detected.

**FIGURE 4:**
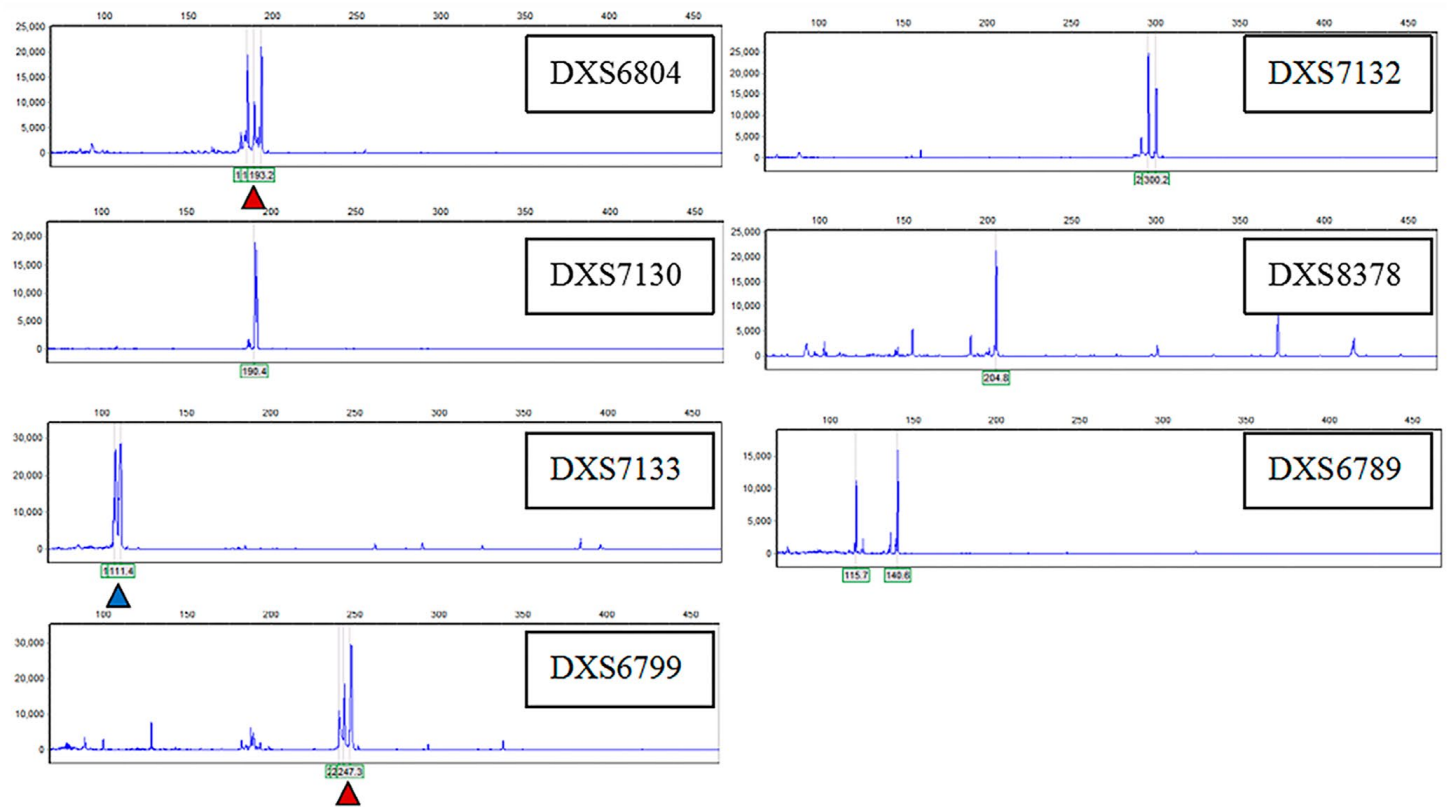
STR analysis demonstrating mosaicism (three peaks at DXS6804/DXS6799). Two of the seven STR loci of chromosome X had three peaks (marked by the red arrow) and one loci had a peak area ratio of about 2:1 (marked by the blue arrow).

Multiplex ligation‐dependent probe amplification (MLPA) using the SALSA P095 kit (MRC‐Holland) confirmed a single copy of Xp22.12 → Xp11.4 (deletion) and duplication of Xp11.4 → Xq28 (Figure 2). Chromosomal microarray (Agilent 8x60K, 0.1 Mb resolution) refined the deletion to 41.25 Mb (ChrX: 2.61–43.86 Mb) and duplication to 111.4 Mb (ChrX: 43.86–155.26 Mb) (Figure 3). Short tandem repeat (STR) analysis of seven X‐chromosome loci revealed three peaks at DXS6804 and DXS6799, indicating mosaicism (Figure 4, Table 1).

**TABLE 1:** The detection of short tandem repeats in chromosome X.

Genetic locus	Fluorescent mark	Location of chromosome	Result of STR	Start position	End position
DXS6804	FAM	Long arm	Three peaks	112112587	112112915
DXS7132	FAM	Long arm	Two peaks	64655267	64655632
DXS7130	FAM	Short arm	One peak	2468039	2468214
DXS8378	FAM	Short arm	One peak	7252119	7252322
DXS7133	FAM	Long arm	Two peaks, ratio of 1:2	109041338	109041858
DXS6789	FAM	Long arm	One peak	95449241	95449556
DXS6799	FAM	Long arm	Three peaks	97378873	97379212

*Note:* Reference system of starting and ending loci [GRCh37].

## 4 Discussion

Turner's syndrome is the most common sex chromosomal abnormality in females, affecting an estimated 3% of all females conceived [5, 13, 14, 15]. It is associated with multiple chromosome abnormalities including 45,X and 45,X/46,XX and 45,X/47,XXX and 45,X/46,XY [1]. It is well known that two basic hypotheses on the manifestation of Turner's syndrome can be considered. Firstly, two X‐chromosomes are necessary for the survival of early human XO conceptuses. Secondly, it is haploinsufficiency or imbalance of the same gene products in the X‐ and Y‐chromosome's nonactivated homologous regions [16].

TS patients are usually overweight, have central obesity, truncal fat mass, short stature, and high BMI, and are at risk for diabetes, which can cause cardiovascular abnormalities [8]. TS is also associated with vascular wall abnormalities that are both structural and functional [17]. Although some factors in the pathogenesis of hypertension remain uncertain, the potential pathogenesis includes inappropriate activation of the renin–angiotensin–aldosterone system, oxidative stress, inflammation, impaired insulin‐mediated vasodilatation, increased stimulation of the sympathetic nervous system, and abnormal sodium processing by the kidney [18, 19, 20, 21, 22, 23].

This case highlights the diagnostic complexity of mosaic Turner syndrome (TS) with structural X anomalies. While 45,X/46,XX mosaicism is well documented, the derivative X chromosome (der(X)) involving breakpoints at Xq13 and Xp11.4 is exceptionally rare. The Xp22.33‐p11.3 deletion encompasses the *SHOX* gene (responsible for short stature), while the Xp11.3‐q28 duplication includes *PLP1* and *TCEAL3/4*, associated with neurodevelopmental risks. Notably, the patient's retained ovarian remnants (immature left ovary) align with reports of partial gonadal function in mosaic TS, suggesting that even minor populations of normal cells may mitigate complete gonadal dysgenesis.

Discordant findings between karyotyping and MLPA underscore the necessity of multimodal diagnostics. While karyotyping identified the der(X), MLPA and chromosomal microarray resolved the breakpoints and dosage imbalances, illustrating how structural rearrangements may evade detection by single‐method approaches. Clinicians must therefore integrate cytogenetic and molecular techniques when managing atypical TS cases.

The patient's management exemplifies the need for lifelong multidisciplinary care. Annual cardiac surveillance (e.g., echocardiography) is critical given the elevated risk of aortic dilation in TS, while hormone replacement therapy (HRT) aims to induce puberty and prevent osteoporosis. Psychological support is equally vital to address body image concerns and psychosocial challenges linked to delayed puberty.

## 5 Conclusion

We used chromosome karyotype analysis, MLPA analysis, 0.1 Mb duplicate or missing detection analysis, and STR analysis, etc. We firstly reported that a 24‐year‐old Chinese female chromosome karyotype is 45,X/46,XX, der(X)(Xqter → Xq13?::Xp11.4 → Xqter), mosaic ratio of 34/166, diagnosed as TS. Clinicians should remember that clinical features and testing items are essential in patients with TS. Furthermore, the possible pathogenic factors of TS should be elucidated in future studies.

## Author Contributions

**Weijun Jiang:** conceptualization, data curation, formal analysis, investigation, methodology, project administration, resources, software, supervision, validation, visualization, writing – original draft, writing – review and editing. **Tingting Ji:** conceptualization, data curation, resources, validation. **Qiuyue Wu:** data curation, formal analysis, investigation, methodology, project administration. **Zhipeng Xu:** resources, writing – review and editing. **Xinyi Xia:** funding acquisition, writing – review and editing.

## Consent

Written informed consent was obtained from the patient to publish this report in accordance with the journal's patient consent policy.

## Conflicts of Interest

The authors declare no conflicts of interest.

## Data Availability

We declared that materials described in the manuscript, including all relevant raw data, will be freely available to any scientist wishing to use them for non‐commercial purposes, without breaching participant confidentiality.
